# Acute cholecystitis and residual choledocholithiasis in a situs inversus patient, successful laparoscopic approach and ERCP a case report from Ecuador

**DOI:** 10.1016/j.amsu.2020.04.012

**Published:** 2020-05-05

**Authors:** Miguel A. Moyon C, Christian L. Rojas, Fernando X. Moyon C, William G. Aguayo, Gabriel A. Molina, Carlos Ochoa, Andres Neira, Maria Elena Vinueza

**Affiliations:** a Department of General Surgery Hospital San Francisco de Quito, IESS Quito, Ecuador; b Department of General Surgery at Hospital IESS Quito Sur Quito, Ecuador; c PGY2 General Surgery Resident, PUCE, Quito, Ecuador; d PGY4 General Surgery Resident, UCE, Quito, Ecuador

**Keywords:** Situs inversus, Acute cholecystitis, Laparoscopic cholecystectomy

## Abstract

**Background:**

Situs inversus totalis is a rare genetic condition characterized by the transposition of organs to the opposite side of the body, consequently, clinical syndromes show an atypical clinical picture creating a challenge for the surgery team and predisposing to delays in treatment and diagnosis. Laparoscopic cholecystectomy is the gold standard for acute cholecystitis, and in patients with situs inversus, the laparoscopic technique must be modified to accommodate the patient's anatomy.

**Case presentation:**

We present the case of a 55-year-old male patient without any past medical history, he presented to the emergency room with abdominal pain in his upper left quadrant. After a thorough examination, acute cholecystitis and situs inversus was diagnosed. He underwent a modified laparoscopic cholecystectomy without complications. In his postoperative period, residual choledocholithiasis was identified and ERCP was done. On follow-ups, the patient is doing well.

**Conclusions:**

Although rare and technically demanding, laparoscopic cholecystectomy and ERCP in a patient with situs inversus is feasible. The altered anatomy could lead to complex procedures, therefore proper planning and careful execution of intraoperative techniques are required to treat these patients safely and effectively.

## Highlights

• Situs inversus totalis is a rare condition characterized by the transposition of organs to the opposite side of the body.
• Laparoscopic cholecystectomy is the gold standard for acute cholecystitis, even in patients situs inversus.
• Proper planning and careful execution of intraoperative techniques are required to treat these patients safely.

## 1 Introduction

This work has been reported in line with the SCARE criteria [14].

Situs inversus is a rare congenital condition characterized by the transposition of organs to the opposite side of the body [1,2]. Patients with this anomaly can present with cholelithiasis and cholecystitis in a similar frequency to that observed in the normal population, nonetheless, signs and symptoms in these patients are misleading due to their altered anatomy [[3], [4], [5]]. When surgery is needed, due to their condition, a more demanding technique and high surgical skills are essential to overcome these rare scenarios [1,3]. We present the case of a 55-year-old male with acute cholecystitis and situs inversus. He underwent a modified laparoscopic cholecystectomy without complications. On follow-ups, the patient is doing well.

## 2 Case report

**Fig. 1:**
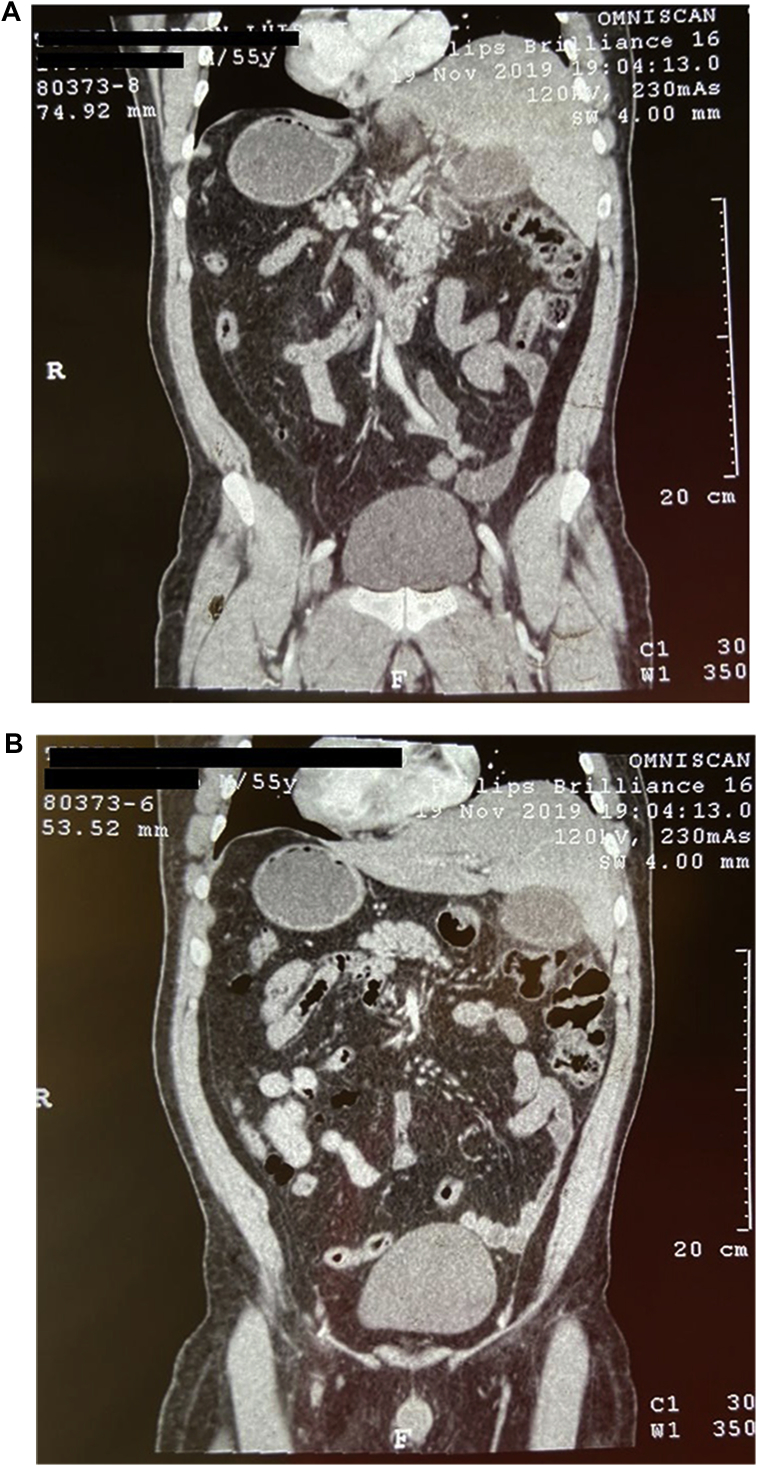
ASitus Inversus, the patient liver is seen on the left side. B: Situs inversus, with cholecystitis.

**Fig. 2:**
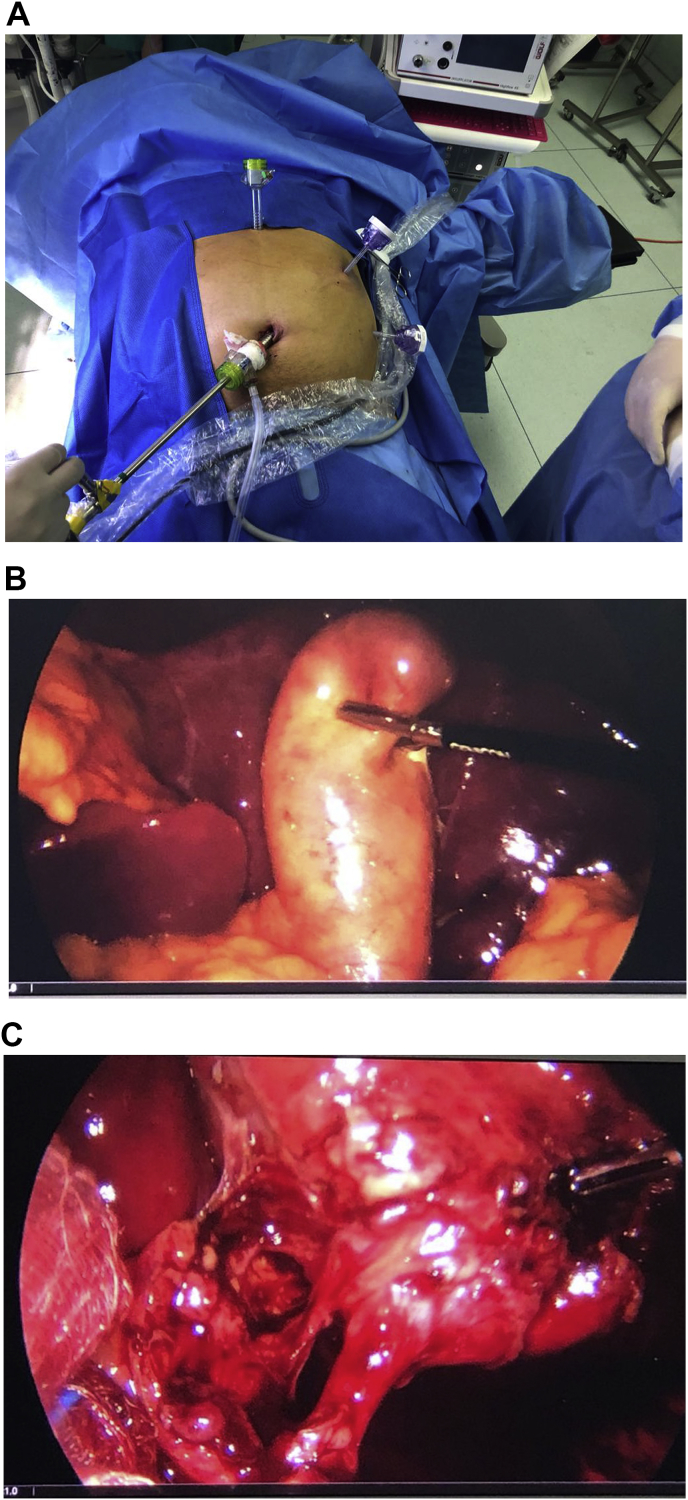
ALaparoscopic Ports positioning. B: Intraoperative view of the gallbladder. C: Elements of the triangle of Calot.

**Fig. 3:**
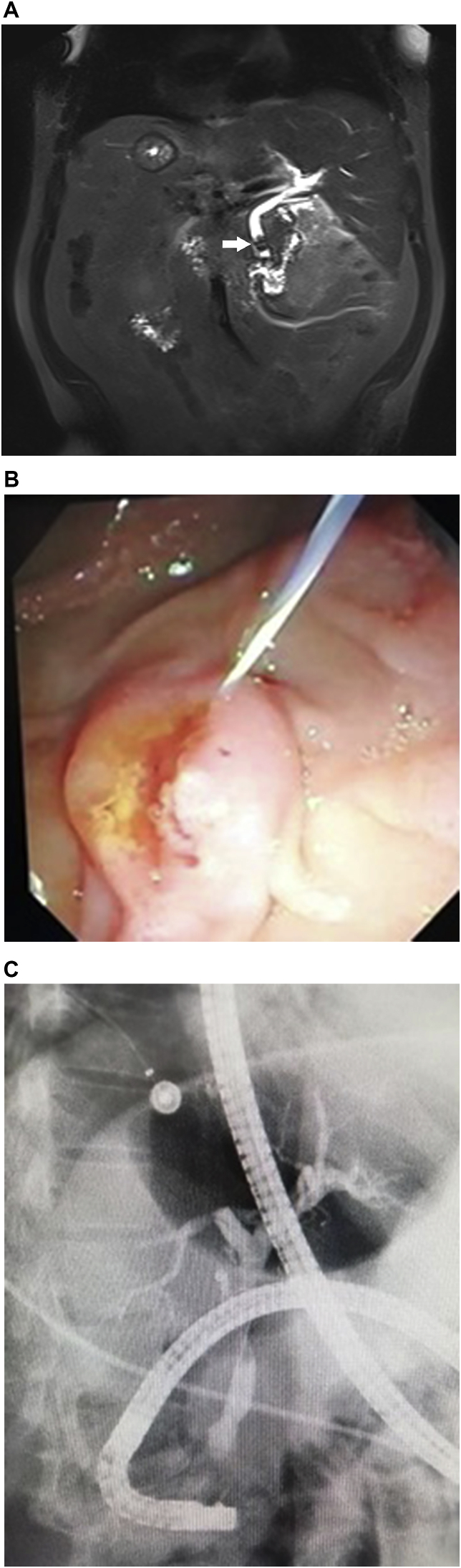
AMRI, revealing residual choledocholithiasis. B: ERCP, Sphincterotomy. C: ERCP, Bile duct without choledocholithiasis.

Patient is a 55-year-old male, without past medical history. He presented to the emergency room with a 3-day history of upper abdominal pain, nausea and vomits. His pain was located on his upper left quadrant and worsened after food intake. On clinical examination, a febrile and dehydrated patient was encountered, he had severe pain on palpation with tenderness in the upper left quadrant. He had no jaundice, weight loss, masses or lymph nodes. Complementary exams revealed leukocytosis, neutrophilia, and an elevated C-reactive protein. His liver and pancreatic exams were normal, ALT 16 U/L, AST 25 U/L, GGT 36 U/L, his total bilirubin was 0.3 mg/dl and lipase was 27 U/L. An abdominal echography was inconclusive and only revealed that the liver was located mainly on the left side of the abdomen with the spleen on the right. Therefore, a contrast-enhanced computed tomography (CT) was requested. Situs Inversus Totalis was detected along with evidence of cholecystitis, the gallbladder had multiple gallstones with wall thickening, pericholecystic stranding, and pericholecystic fluid. (Fig. 1A and B). A magnetic resonance image (MRI) was requested to assess the biliary anatomy and for preoperative planning. On his preoperative CT and MRI, the bile duct was clear of bile duct stones and was not dilated. The patient was scheduled for laparoscopic cholecystectomy by the surgical department; however, several adjustments were done to perform this unusual surgery. The monitor was placed beside the head of the patient on the left side, the surgeon and the assistants stood on the right side. We placed a 10mm transumbilical port for the camera, a 10mm epigastric port, and two 5 mm port in the left side of the abdomen; one at the left midclavicular line and another at the anterior axillary line for traction of the fundus of the gallbladder (Fig. 2A). The gallbladder had thickened walls and had several adhesions to the omentum. The infundibulum was grasped with the right hand of the surgeon and the dissection was achieved using electrocautery with the left hand of the surgeon. After the critical view of safety of the triangle of Calot was achieved, the cystic duct and artery were clipped, and the rest procedure was completed without complications (Fig. 2B and C). His postoperative period was uneventful, a liquid diet was initiated on his first postoperative day and the patient was discharged without complications. Nonetheless, twenty days after laparoscopic cholecystectomy the patient presented to the emergency room. This time he had jaundice, fever, and abdominal pain. Complementary exams revealed cholestasis, with bilirubin levels over 12 mg/dl. A new MRI was requested revealing residual choledocholithiasis (Fig. 3A), an endoscopic retrograde cholangio pancreatography (ERCP) was planned. The stone was removed from the bile duct and the patient successfully recovered (Fig. 3B and C). On follow-ups, the patient is doing well without any complications.

## 3 Discussion

Situs inversus (SI) is a rare autosomal recessive genetic condition in which organs are shifted from their regular positions to locations on the opposite side of the body [1,2]. This condition can affect thoracic or abdominal organs [2,3]. When this condition affects both organs it is named situs inversus totalis, this rare condition affects 1 in every 5.000 to 20.000 people and was first described by Fabricius in 1600 [1,3]. As organs are in an anatomically modified position, they can cause diagnostic challenges in many pathologies [4]. As it was found in our patient. The transposition of the organs can be associated with many congenital anomalies including biliary atresia, renal dysplasia, congenital heart disease, pancreatic fibrosis, among others [1,5]. Although situs inversus does not influence gallbladder disease it can lead to diagnostic and surgical difficulties [6].

Clinical manifestations apart from the left abdominal pain are similar to those occurring in typical right-sided gallbladders [1,7]. SI can be suspected when there is a beat in the right fifth intercostal space or dullness on the left side of the body, nonetheless, ultrasound, CT, or MRI can confirm the diagnosis [8]. In our patient, upper left pain was found, and a CT confirmed the diagnosis. Cholecystitis in the setting of SI is a unique condition that has been reported in less than 50 cases in the literature. Surgery is the treatment of choice, nonetheless, the technical difficulty of the inverted anatomy requires meticulous surgical techniques to avoid complications [1,9]. Conventional open approach was the traditional surgical technique until 1991 when Campos and Sipes reported the first laparoscopic approach in an SI patient [10]. This is the first-ever reported case with a laparoscopic approach in Ecuador.

Laparoscopic approach in an SI patient requires complete knowledge of biliary anatomy [1,11]. The ports positioning needs to change to suit the patient's anatomy and right-handed surgeons may find difficulty handling the gallbladder during the dissection [1,4,12]. As vascular and biliary anomalies are more common in patients with SI, an intraoperative cholangiogram or a preoperative MRI can aid during surgery [1,3]. As it was done in our patient. Nonetheless, achieving the critical view of safety and carefully identifying the anatomy in Calot's triangle should be achieved every time [1,13]. If ERCP is required sphincterotomy and common bile duct extraction can be complex and requires skillful management and adaptability from the endoscopist [12,13]. In our case, surgery was completed without complications, nevertheless, residual choledocholithiasis was detected in his postoperative period and was successfully managed with an ERCP.

Laparoscopic cholecystectomy and ERCP in a patient with situs inversus are feasible if adequate laparoscopic and endoscopic equipment is available along with trained and experienced surgeons and endoscopists. Since the surgical technique is more demanding, preoperative planning and careful dissection can dramatically improve patients’ outcomes and prevent dangerous complications.

## Ethical approval

We have approval from the surgical department and ethics committee from IESS hospital.

## Sources of funding

None.

## Author contribution

1. Miguel A. Moyon C. writing the paper, data analysis or interpretation.

2. Christian L. Rojas MD, data analysis or interpretation.

3. F. Xavier Moyon C. MD, data analysis or interpretation.

4. William G. Aguayo MD, data analysis or interpretation.

5. Gabriel A. Molina MD, study concept or design, writing the paper.

6. Carlos Ochoa MD data collection.

7. Andres Neira MD, study concept or design.

8. Maria Elena VinuezaMD, data collection.

## Registration of research studies

This is a case report, no human participants were involved.

## Guarantor

Gabriel A. Molina MD, Department of General Surgery at Hospital IESS Quito Sur Quito-Ecuador, Attending surgeon, gabomolina32@gmail.com, + 593 998352535, Grla. Eloy Alfaro S6 119 y Pasaje Cueva, San Juan de Cumbaya.

## Consent

We have signed consent from the patient.

## Provenance and peer review

Not commissioned, externally peer reviewed.

## Declaration of competing interest

The authors declares that no relevant or material financial interests exists.
